# WTAP promotes myocardial ischemia/reperfusion injury by increasing endoplasmic reticulum stress via regulating m^6^A modification of ATF4 mRNA

**DOI:** 10.18632/aging.202770

**Published:** 2021-03-26

**Authors:** Jiayi Wang, Jiehan Zhang, Yan Ma, Yuxiao Zeng, Cheng Lu, Fenghua Yang, Nianxin Jiang, Xuan Zhang, Yuhua Wang, Yinghui Xu, Hanjing Hou, Shengyang Jiang, Shaowei Zhuang

**Affiliations:** 1 Department of Cardiology, Seventh People’s Hospital of Shanghai University of Traditional Chinese Medicine, Shanghai 200137, China

**Keywords:** myocardial infarction, I/R injury, endoplasmic reticulum stress, m^6^A modification, Wilms' tumor 1-associating protein

## Abstract

Myocardial infarction (MI) is one of the leading causes of death. Wilms' tumor 1-associating protein (WTAP), one of the components of the m^6^A methyltransferase complex, has been shown to affect gene expression via regulating mRNA modification. Although WTAP has been implicated in various diseases, its role in MI is unclear. In this study, we found that hypoxia/reoxygenation (H/R) time-dependently increased WTAP expression, which in turn promoted endoplasmic reticulum (ER) stress and apoptosis, in human cardiomyocytes (AC16). H/R effects on ER stress and apoptosis were all blocked by silencing of WTAP, promoted by WTAP overexpression, and ameliorated by administration of ER stress inhibitor, 4-PBA. We then investigated the underlying molecular mechanism and found that WTAP affected m^6^A methylation of ATF4 mRNA to regulate its expression, and that the inhibitory effects of WTAP on ER stress and apoptosis were ATF4 dependent. Finally, WTAP’s effects on myocardial I/R injury were confirmed *in vivo*. WTAP promoted myocardial I/R injury through promoting ER stress and cell apoptosis by regulating m^6^A modification of ATF4 mRNA. These findings highlight the importance of WTAP in I/R injury and provide new insights into therapeutic strategies for MI.

## INTRODUCTION

Myocardial infarction (MI), also known as heart attack, occurs when blood flow decreases or stops suddenly, leading to death of heart muscle [1]. It has been shown that MI affects about one million people per year in the United States alone [1, 2]. A common pathophysiological feature of MI is ischemia and reperfusion (I/R) injury [3]. Previous research has found that many changes, including apoptosis and endoplasmic reticulum (ER) stress, occur during reperfusion after ischemia [4]. Apoptosis has been shown to be an ongoing process during ischemia and is boosted by reperfusion, which not only restores oxygen and glucose supply for viable cells but also provides energy for the completion of apoptosis [4, 5]. A previous study indicated that I/R promoted caspase 3 activation and apoptosis [6]. Poly(ADP-ribose) polymerase (PARP) has been shown to be cleaved to trigger apoptosis by heart I/R injury [7]. C/EBP homologous protein (CHOP) has been implicated in ER stress–induced apoptosis signaling pathways and the up-regulation of CHOP serves as a symbol of the PERK signal pathway activation [8–10]. Increasing evidence also shows that ER stress contributes to I/R-induced damage [11]. The stress in the ER activates Unfolded Protein Response (UPR) pathways via the induction of protein kinase RNA-like endoplasmic reticulum kinase (PERK) [12].

In addition, ER stress can transcriptionally activate a number of adaptive pathways that are tightly controlled by a family of stress-responsive transcription factors including the activating transcription factor 4 (ATF4) [13], which controls expression of ER stress-related genes [14]. Studies have shown that ATF4 could be regulated by a variety of factors, including hypoxic stress and phosphorylation of eukaryotic translation initiation factor 2α (eIF2α) [15, 16]. Recent study also demonstrated that N^6^-methyladenosine (m^6^A), the most prevalent internal modification on the messenger RNA (mRNA) of all higher eukaryotes, also regulates ATF4 expression through modulating its mRNA stability [17, 18]. The m^6^A formation is catalyzed by the methyltransferase like 3 (METTL3)-containing methyltransferase complex [19]. Wilms' tumor 1-associating protein (WTAP) has been shown to regulate recruitment of the m^6^A methyltransferase complex to mRNA targets [19]. Although WTAP is implicated in various biological processes such as eye development and proliferation of vascular smooth muscle cells [20–22], its role in MI remains unclear. Therefore, in this study, the role of WTAP and its effects on m^6^A modification of ATF4 mRNA, ER stress, and apoptosis in MI were investigated.

## RESULTS

### WTAP knockdown inhibited H/R-induced cell apoptosis and ER stress in AC16 cells

**Figure 1:**
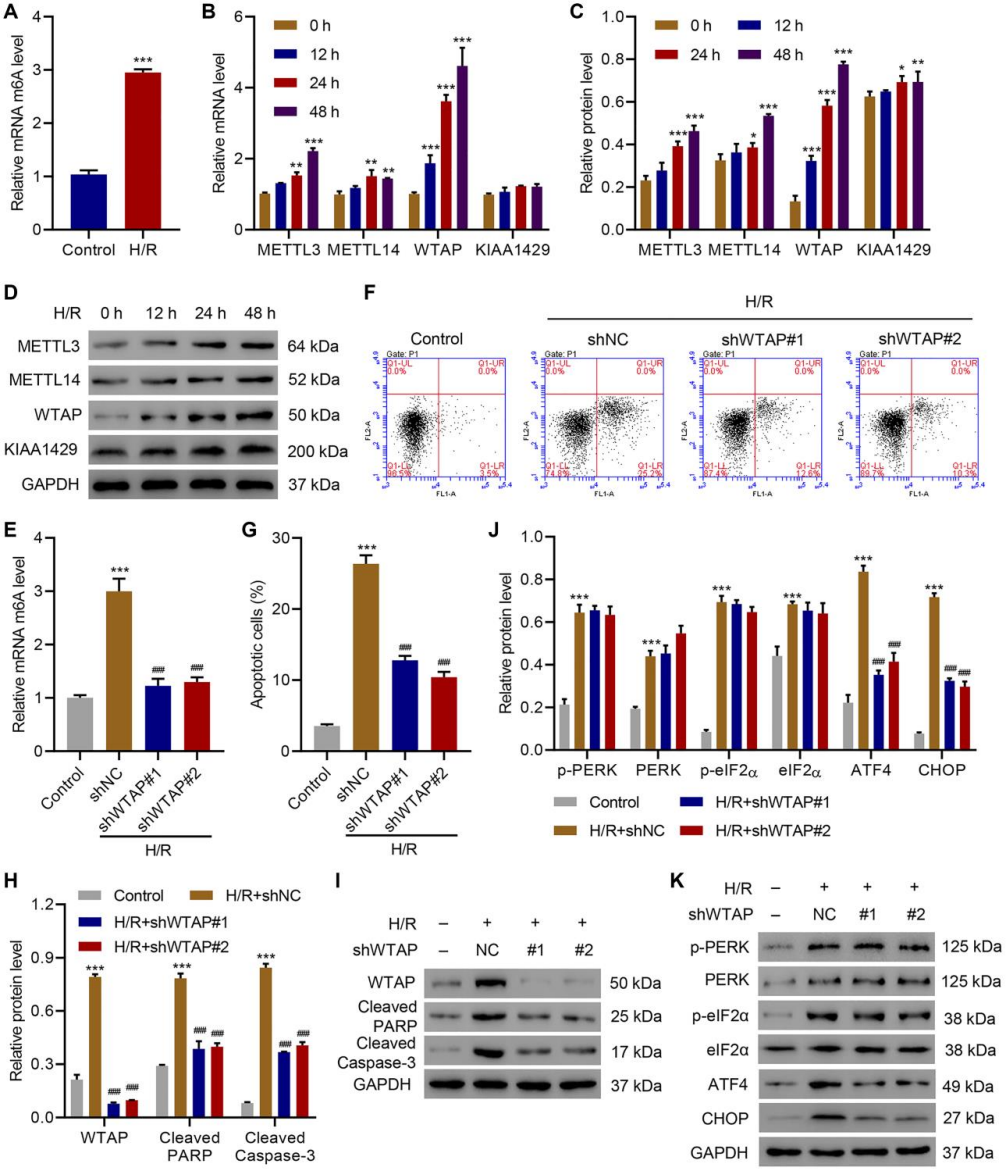
**WTAP knockdown inhibits H/R-induced injury.** (**A**) The m^6^A levels in H/R AC16 cells. (**B–D**) Expression of METTL3, METTL14, WTAP and KIAA1429 in AC16 cells after H/R treatment for indicated time courses. (**E**) The m^6^A levels in AC16 cells transduced with WTAP shRNA and treated with H/R for 48 h were measured by ELISA. (**F, G**) Cell apoptosis and (**H–K**) protein levels of WTAP, cleaved PARP, Cleaved Caspase-3, p-PERK, PERK, p-eIF2α, eIF2α, ATF4 and CHOP in AC16 cells transduced with WTAP shRNA and treated with H/R for 48 h were measured by flow cytometry and Western blotting, respectively. All experiments were repeated at least three times, and data are represented as mean ± SD. ^*^P < 0.05, ^**^P < 0.01, ^***^P < 0.001 vs control (untreated) or 0 h. ^###^*P* < 0.001 vs H/R+shNC.

In order to investigate the role of WTAP in apoptosis and ER stress, we first checked the effect of H/R on m^6^A levels. Data suggested H/R significantly up-regulated m^6^A compared with that of controls (Figure 1A). Because previous study showed that in addition to WTAP, other methyltransferases including methyltransferase like 3 (METTL3), METTL14, and vir-like m^6^A methyltransferase associated (VIRMA, KIAA1429) also regulates m^6^A modification, so we measured the expression of METTL3, METTL14, WTAP and KIAA1429 [23]. Results showed that at the mRNA level, H/R time-dependently increased the expression of METTL3, METTL14, WTAP, but did not affect the expression of KIAA1429 (Figure 1B). Western blotting results showed that H/R time-dependently increased the expression of WTAP at the protein level, but not the expression of METTL3, METTL14 and KIAA1429 (Figure 1C, 1D). Then, WTAP was silenced in AC16 cells (Supplementary Figure 1) underwent H/R. ELISA results showed that silencing of WTAP significantly suppressed H/R-caused elevation of m^6^A levels (Figure 1E). Flow cytometry analysis showed that silencing of WTAP significantly suppressed H/R-induced apoptosis (Figure 1F, 1G). Then western blots were performed to check the effects of WTAP silencing on the expression of apoptotic marker proteins and ER stress marker proteins. Results showed that silencing of WTAP significantly inhibited H/R-induced upregulation of cleaved PARP and cleaved Caspase-3 (Figure 1H, 1I). Silencing of WTAP also significantly inhibited H/R-induced upregulation of ATF4 and CHOP, but did not show significant effect on H/R-induced upregulation of ER-stress related marker proteins including p-PERK, PERK, p-eIF2α, eIF2α (Figure 1J, 1K). The findings indicated H/R elevated m^6^A levels, promoted apoptosis and ER stress in AC16 cells through upregulating WTAP.

### 4-PBA protected AC16 cells from WTAP overexpression-induced apoptosis and ER stress

**Figure 2:**
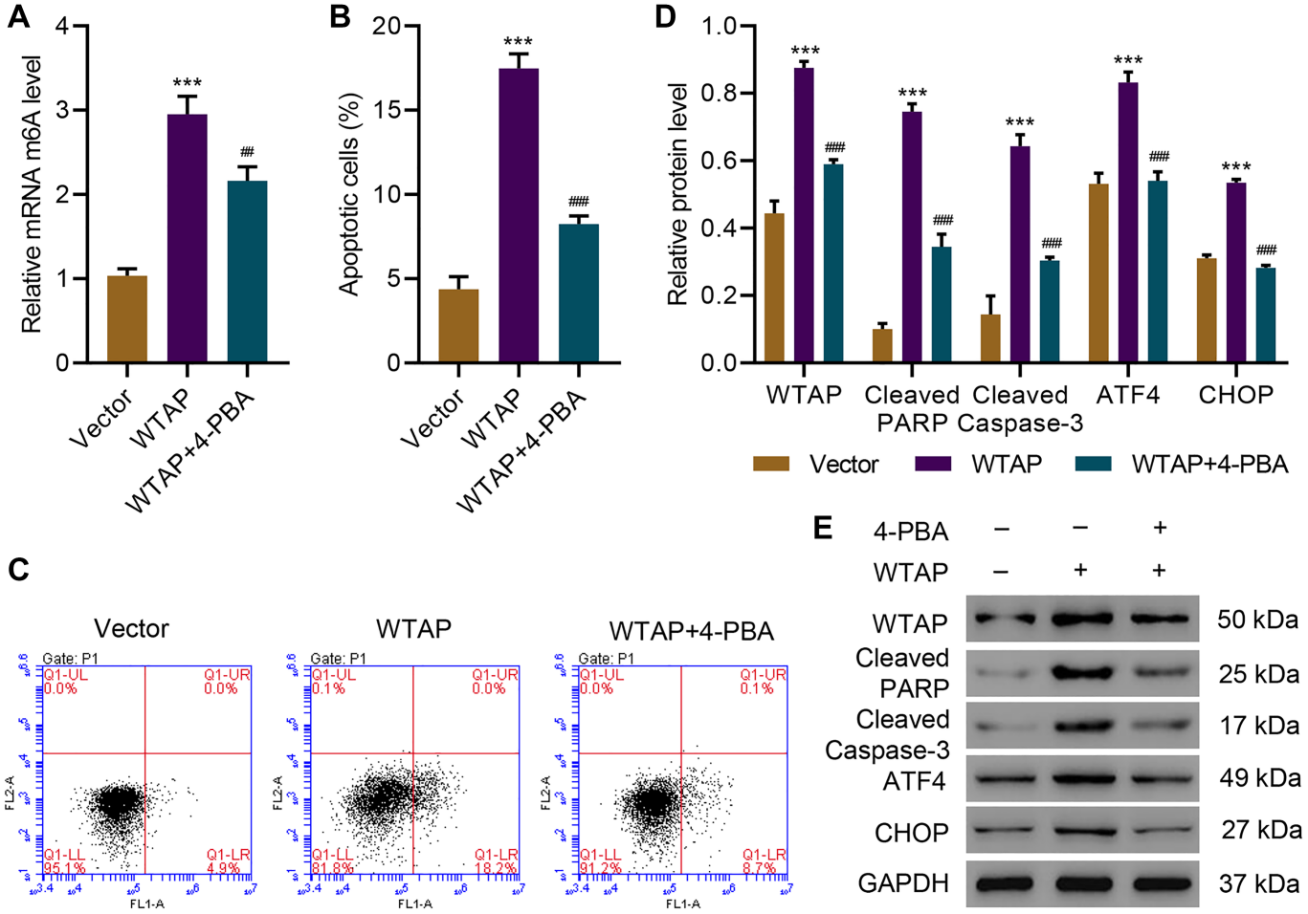
**4-PBA protects AC16 cells from apoptosis and endoplasmic reticulum stress induced by WTAP overexpression.** (**A**) The m^6^A levels in AC16 cells transduced with WTAP-overexpressing plasmid and treated with 2 mM 4-PBA for 48 h were measured by ELISA. (**B, C**) Cell apoptosis and (**D, E**) expression of WTAP, PARP, cleaved Caspase-3, ATF4 and CHOP. All experiments were repeated at least three times, and data are represented as mean ± SD. ^***^P < 0.001 vs vector. ^##^*P* < 0.01, ^###^*P* < 0.001 vs WTAP.

To further investigate the role of WTAP in ER stress and cell apoptosis, WTAP was successfully overexpressed in AC16 cells (Supplementary Figure 2) and WTAP-overexpressing AC16 cells were treated with an ER stress inhibitor, 4-PBA (2 mM, 48 h). Overexpression of WTAP significantly increased m^6^A level, while this effect was diminished by administration of 4-PBA (Figure 2A). Next, the effects of WTAP overexpression on ER stress and apoptosis were evaluated. Overexpression of WTAP remarkably increased apoptosis, which was suppressed by administration of 4-PBA (Figure 2B, 2C). Western blots indicated that overexpression of WTAP enhanced levels of cleaved-PARP, cleaved Caspase-3, ATF4 and CHOP, which was abolished by administration of 4-PBA (Figure 2D, 2E). These findings suggested that WTAP-mediated ER stress resulted in cell apoptosis indicated by blockage of apoptosis by administration of an ER stress inhibitor.

### WTAP knockdown inhibited H/R-induced injury in AC16 cells by suppressing ATF4

**Figure 3:**
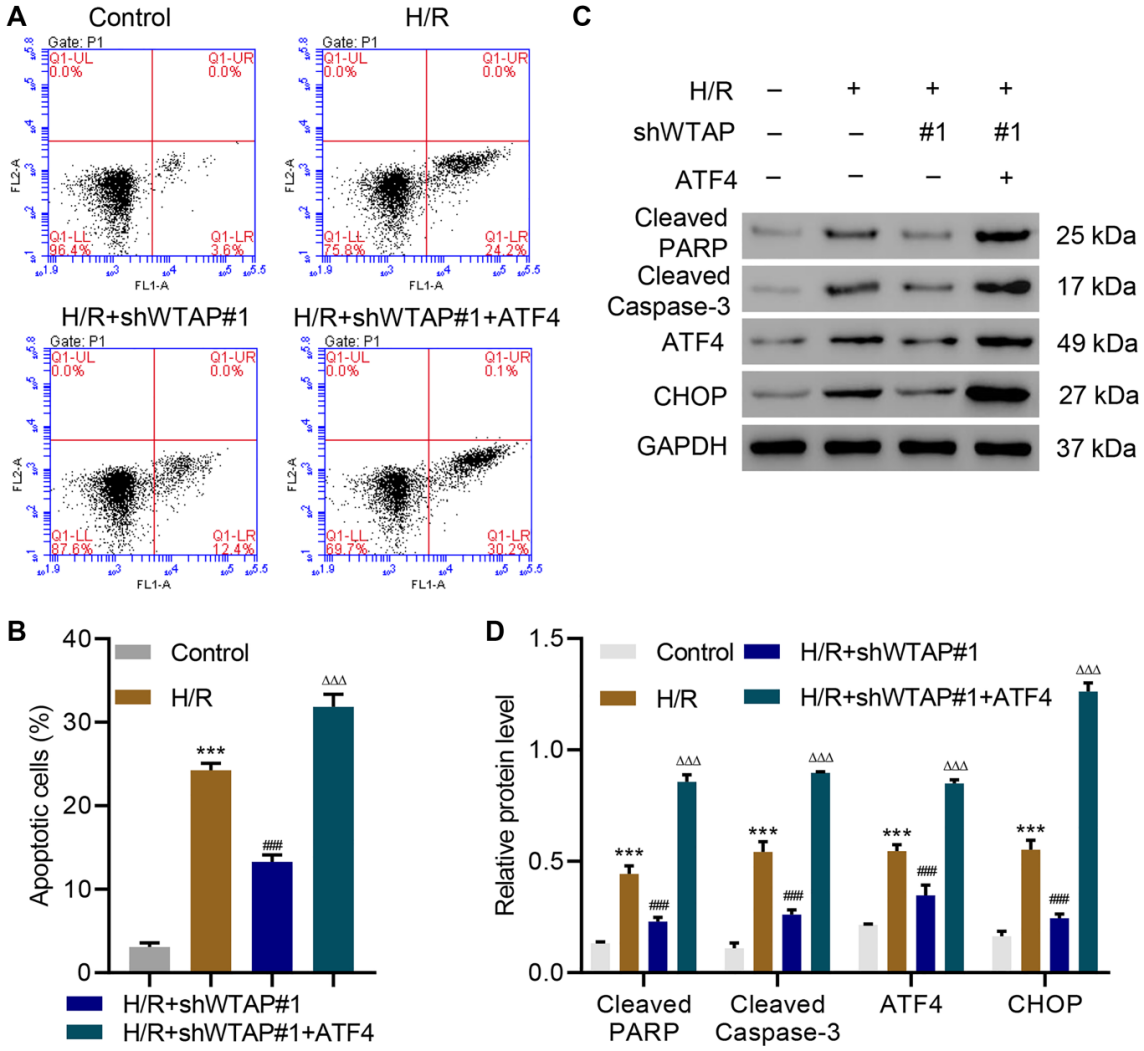
**WTAP knockdown inhibits H/R injury in AC16 cells by suppressing ATF4.** (**A, B**) Cell apoptosis and (**C, D**) expression of PARP, cleaved Caspase-3, ATF4 and CHOP in AC16 cells transduced with WTAP shRNA and ATF4-overexpressing plasmid. All experiments were repeated at least three times, and data are represented as mean ± SD. ^***^P < 0.0001 compared with control (untreated). ^###^*P* < 0.001 vs H/R. ^ΔΔΔ^*P* < 0.001 vs H/R+shWTAP#1.

To explore how WTAP silencing inhibits H/R-induced injury, ATF4 was successfully overexpressed in WTAP-silenced AC16 cells (Supplementary Figure 2) and the cells underwent H/R. Flow cytometry analysis showed that WTAP silencing significantly inhibited H/R-induced apoptosis, which was reversed by overexpression of ATF4 (Figure 3A, 3B). Western blots confirmed that WTAP silencing significantly inhibited H/R-induced expression of cleaved PARP, cleaved Caspase-3, ATF4 and CHOP, which was also reversed by overexpression of ATF4 (Figure 3C, 3D). The findings showed WTAP knockdown inhibited H/R-induced injury in AC16 through suppression of ATF4.

### WTAP targeted ATF4 in H/R-induced injury of AC16 cells

**Figure 4:**
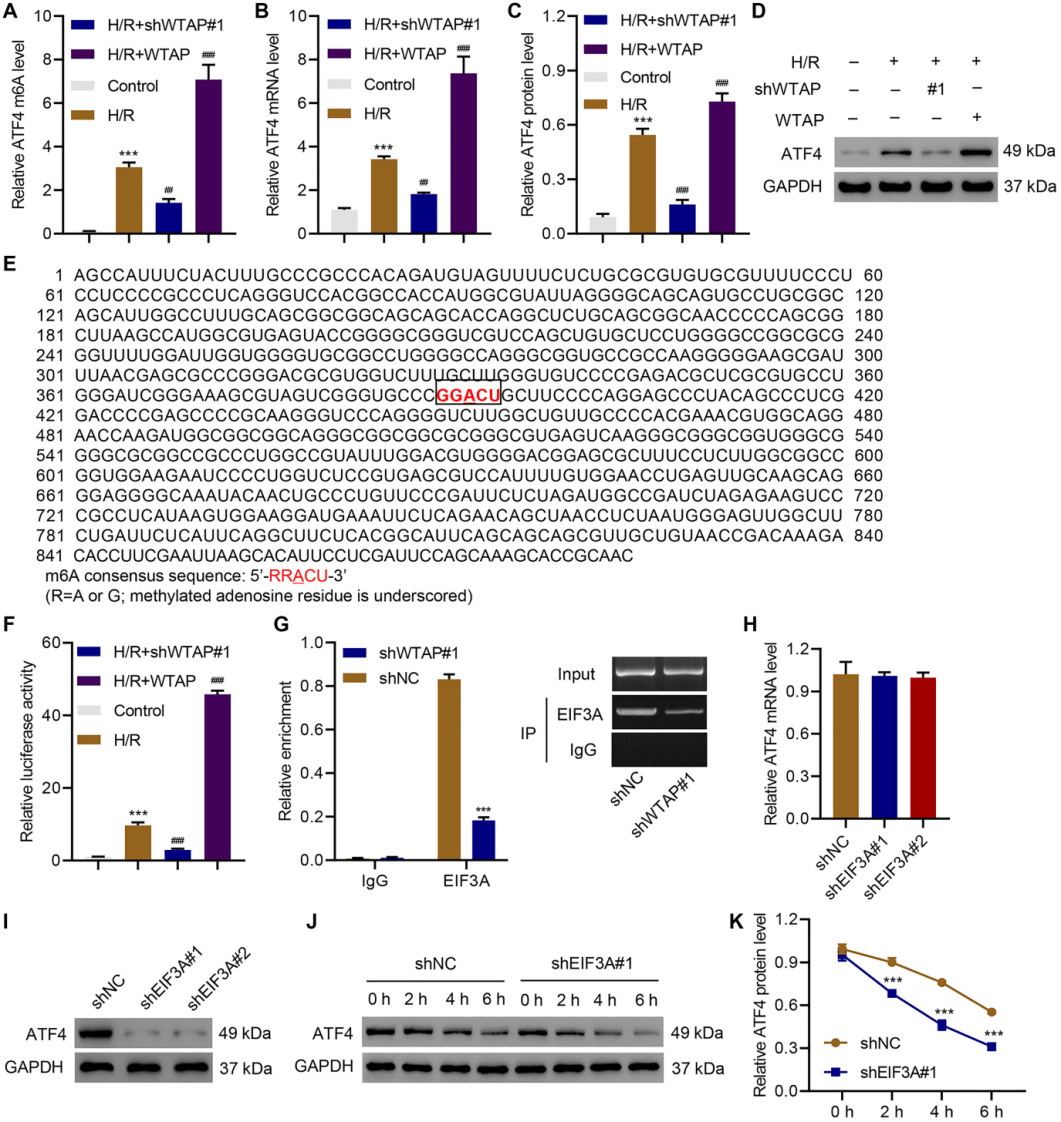
**Identification of ATF4 as a target of WTAP.** AC16 cells were transduced with WTAP shRNA or WTAP-overexpressing plasmids and underwent H/R for 48 h. (**A**) MeRIP-qPCR analysis of ATF4 5′UTR m^6^A levels. (**B–D**) Expression of ATF4. (**E**) Analysis of TFEB 5′UTR showed a match to 5′-RRACU-3′ m^6^A consensus sequence. (**F**) Luciferase activity assay. (**G**) Binding of EIF3A to ATF4 mRNA was measured by RIP and qRT-PCR. (**H, I)** The ATF4 expression in AC16 cells transduced with EIF3A shRNAs. (**J, K**) Western blot analysis of ATF4 protein level upon CHX treatment in AC16 cells transduced with EIF3A shRNA. All experiments were repeated at least three times, and data are represented as mean ± SD. ^***^P < 0.001 vs control (untreated)/shNC. ^##^*P* < 0.01, ^###^*P* < 0.001 vs H/R.

To understand how ATF4 is involved in the WTAP regulation of H/R-induced injury, we first analyzed ATF4 5′UTR m^6^A levels in control cells, WTAP-silencing AC16 cells, and WTAP-overexpressing AC16 cells with or without H/R. Results showed that silencing of WTAP significantly inhibited H/R-induced ATF4 5′UTR m^6^A levels. In contrast, overexpression of WTAP sharply increased H/R-induced ATF4 5′UTR m^6^A levels (Figure 4A). qPCR and immunoblotting results suggested silencing of WTAP significantly downregulated ATF4 while overexpression of WTAP significantly upregulated ATF4 (Figure 4B–4D). Then, we did a bioinformatic analysis and found a potential bind site of EIF3A in ATF4-5′UTR (Figure 4E). Therefore, we performed luciferase assay of EIF3A and ATF4-5′UTR interaction. Results showed that silencing of WTAP significantly decreased H/R-induced increase in luciferase activity of ATF4-5′UTR while overexpression of WTAP further increased luciferase activity of ATF4-5′UTR in H/R-treated cells (Figure 4F). We then performed RIP and qRT-PCR and confirmed that EIF3A directly binds to ATF4 mRNA (Figure 4G). To verify these results, EIF3A was successfully silenced in AC16 cells (Supplementary Figure 1). Silencing of EIF3A did not affect the expression of ATF4 at the mRNA level (Figure 4H), but significantly decreased the protein level of ATF4 (Figure 4I). Then, control cells or EIF3A-silencing cells were treated with CHX (100 μg/ml), a protein synthesis inhibitor, to evaluate the effect of EIF3A on protein stability of ATF4. Results showed that silencing of EIF3A time-dependently decreased ATF4 protein level (Figure 4J, 4K). The data indicated WRAP directly targets ATF4.

### ATF4 regulated the expression of WTAP at the transcription level

**Figure 5:**
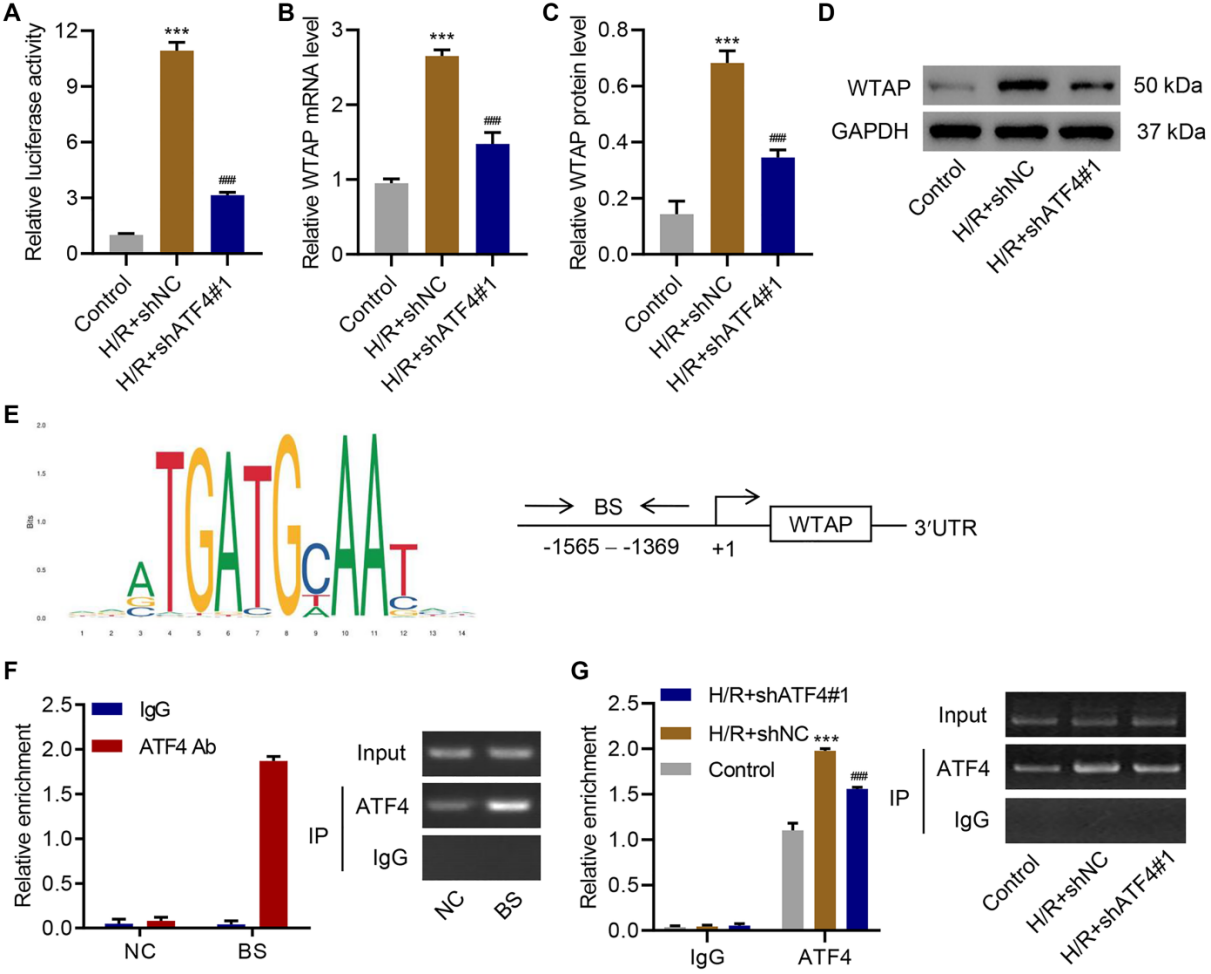
**ATF4 regulates the transcription of WTAP. AC16 cells were transduced with ATF4 shRNA and treated with H/R for 48 h.** (**A**) WTAP promoter activity and (**B–D**) WTAP expression. (**E**) ATF4 binding site (BS) in WTAP promoter schematic diagram. (**F**) Chromatin immunoprecipitation assay of ATF4 binding with WTAP. (**G**) Chromatin fragments from AC16 cells transduced with ATF4 shRNA and H/R were immunoprecipitated to analyze the binding of ATF4 to WTAP. All experiments were repeated at least three times, and data are represented as mean ± SD. ^***^P < 0.001 vs control (untreated). ^###^*P* < 0.001 vs H/R+shNC.

To further investigate the relationship between ATF4 and WTAP, ATF4 was successfully silenced in AC16 cells (Supplementary Figure 1) and the cells underwent H/R treatment. Luciferase reporter assay revealed that silencing of ATF4 significantly inhibited H/R-enhanced WTAP promoter activity (Figure 5A). Silencing of ATF4 also significantly inhibited H/R-increased WTAP mRNA (Figure 5B) and protein levels (Figure 5C, 5D). To understand how ATF4 regulated WTAP expression, we used JASPAR algorithm to analyze WTAP promoter region and found a potential binding site of ATF4 in the promoter region of WTAP (Figure 5E). ChIP assay results confirmed that ATF4 directly interacts with WTAP promoter (Figure 5F). H/R treatment promoted the binding of ATF4 to WTAP promoter, while silencing of ATF4 inhibited this binding (Figure 5G). Taken together, these findings suggested that ATF4 bound to the promoter region of WTAP to regulate its transcription.

### WTAP knockdown reduced cardiac I/R injury *in vivo*

**Figure 6:**
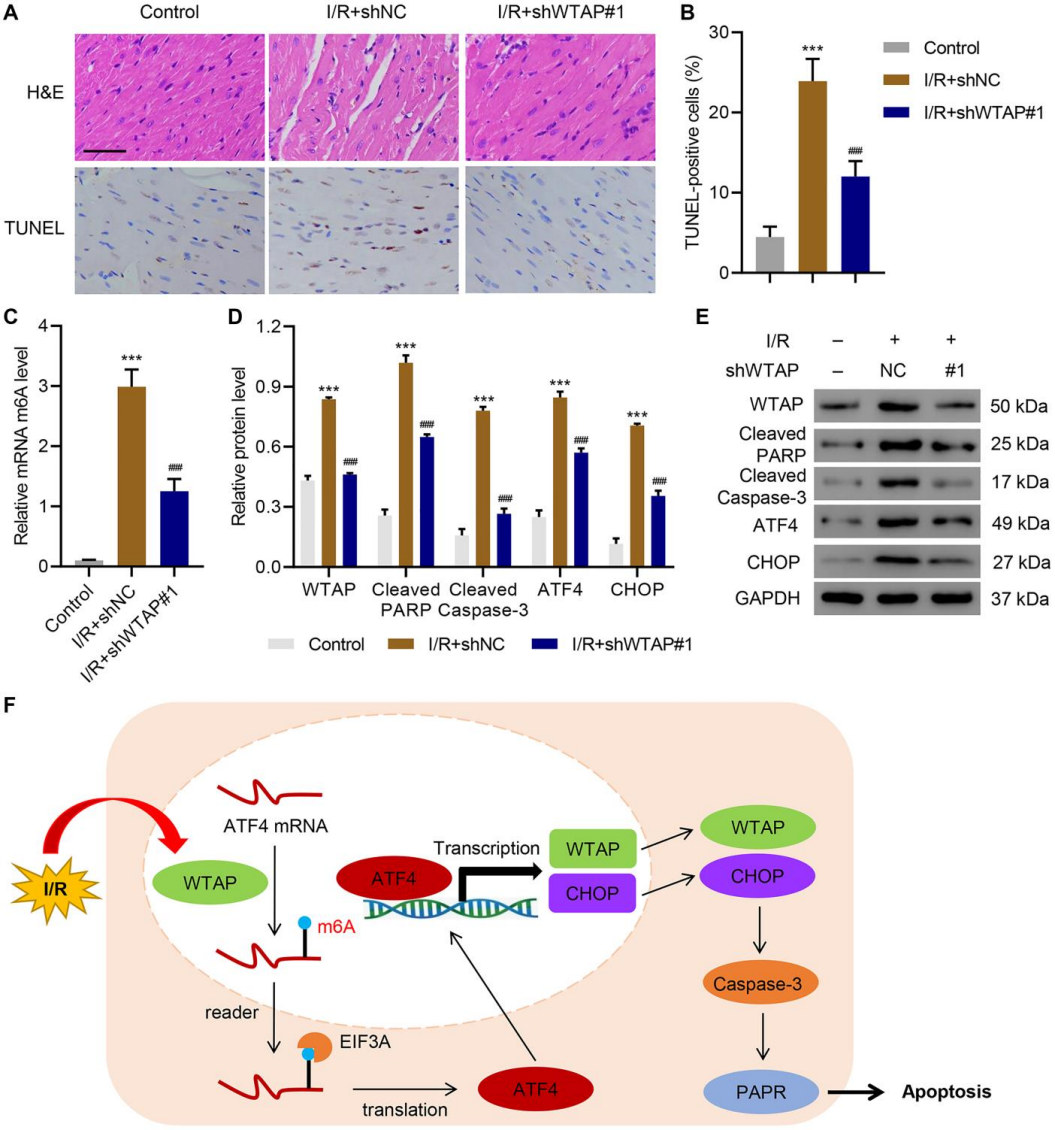
**WTAP knockdown inhibits I/R injury *in vivo*.** WTAP shRNA or negative control (shNC) was injected into rats. (**A, B**) H&E and TUNEL staining analysis of myocardium of rats. Scale bar: 50 μm. (**C**) The m^6^A levels in myocardium of rats were measured by ELISA. (**D, E**) Expression levels of WTAP, cleaved PARP, cleaved Caspase-3, ATF4 and CHOP in myocardial cells of rats. (**F**) Schematic diagram of the relationships among WTAP, m^6^A modification, and cell apoptosis under I/R condition. All experiments were repeated at least three times, and data are represented as mean ± SD. ^***^P < 0.0001 vs control (without LAD ligation). ^###^*P* < 0.001 vs I/R+shNC.

After injecting WTAP shRNA vector or its negative control (shNC) to the rats, cardiac function indexes are shown in Table 1. I/R significantly decreased ejection function (EF), fractional shortening (FS), and systolic blood pressure, which was reversed by WTAP knockdown. After I/R, the myocardium was harvested. HE staining and TUNEL staining showed that silencing of WTAP significantly ameliorated I/R induced myocardial cell apoptosis (Figure 6A, 6B). ELISA results suggested that silencing of WTAP decreased I/R-induced m^6^A levels (Figure 6C). Silencing of WTAP also reduced I/R-induced upregulation of cleaved PARP, cleaved Caspase-3, ATF4 and CHOP in myocardial cells (Figure 6D, 6E). These results indicated that WTAP knockdown reduced cardiac I/R injury *in vivo*.

**Table 1:** Rat cardiac function indexes.

**Group (*n* = 6)**	**Echocardiographic data (%)**		**Blood pressure (mmHg)**	
	**EF**	**FS**	**Diastolic**	**Systolic**
Control	86.86 ± 1.40	49.21 ± 1.84	5.47 ± 0.59	134.0 ± 3.35
I/R + shNC	63.07 ± 2.24^***^	28.28 ± 1.45^***^	13.02 ± 0.62^***^	96.82 ± 2.02^***^
I/R + shWTAP#1	74.06 ± 2.94^###^	36.31 ± 2.52^###^	9.23 ± 0.83^###^	113.5 ± 5.46^###^

Abbreviations: EF, ejection fraction; FS, fractional shortening. ***P < 0.001 compared with control group; ^###^*P* < 0.001 compared with I/R + shNC group.

## DISCUSSION

The present study demonstrated that knockdown of WTAP significantly inhibited H/R-induced injury. In contrast, overexpression of WTAP significantly increased apoptosis and ER stress, which were ameliorated by administration of 4-PBA. In terms of molecular mechanism, we showed that WTAP knockdown protected cardiomyocytes against apoptosis and ER stress through down-regulating the mRNA stability of ATF4. This study also indicated that ATF4 could bind to the promoter region of WTAP to regulate its transcription. Finally, using animal models, we demonstrated that knockdown of WTAP significantly reduced H/R-induced injury *in vivo*.

As one of the most frequently occurring forms of methylation in eukaryotic mRNA, m^6^A modification is catalyzed by a methyltransferase complex containing methyltransferase like 3 (METTL3), methyltransferase like 14 (METTL14) and WTAP [19]. The stability of m^6^A-containing mRNAs is controlled by crosstalk between m^6^A and other cellular factors [24]. A previous study has shown that m^6^A modification was involved in ATF4 regulation [25]. Here we showed for the first time that H/R significantly enhanced WTAP, which is involved in the m^6^A of modification of ATF4 mRNA, thereby regulating ATF4 expression. Overexpression of WTAP resulted in increase of ATF4 probably through enhancing ATF4 mRNA stability. We also found that, on one hand, WTAP directly targets ATF4, on the other hand, ATF4 regulates the transcription of WTAP. These findings revealed a new role of WTAP in MI, showing that WTAP promotes myocardial I/R injury via a positive feedback loop with the transcription factor ATF4.

Apoptosis and ER stress have been implicated in the reperfusion of the myocardium after ischemic damage. ER has been implicated in protein folding and trafficking [26]. ER stress occurs when its function is altered [27]. ER stress has been implicated in the pathogenesis of a variety of diseases including Diabetes mellitus (DM), viral infection, Retinitis pigmentosa (RP) and MI [28]. During ER stress, ATF4 activates a bunch of genes involved in various adaptive pathways [29]. Apoptosis, a form of programmed cell death, is another major pathological process during MI [30]. It has been shown that apoptosis plays a role in the process of cardiomyocyte death [31]. In this study, we showed that overexpression of WTAP promoted both apoptosis and ER stress, while silencing of WTAP inhibited H/R injury via suppression of ATF4. These findings not only increase our knowledge of WTAP in apoptosis and MI, but also broaden our understanding of the pathogenesis of MI.

WTAP function was further studied in a rat model. Left ventricular anterior wall injection of WTAP shRNA significantly inhibited m^6^A modification, ER stress and cell apoptosis under I/R condition, which confirmed the *in vitro* results. The findings indicate a crucial role of WTAP in MI and thus improve our understanding of the roles of WTAP and ATF4 in the pathogenesis of MI. There are certainly some limitations in this study. For example, this study was mainly performed in animal and cell models. Further studies using clinical samples will provide more persuasive data. Although further experiment is needed, this study identifies a new molecular mechanism underlying MI and provides us a new potential therapeutic approach for H/R-induced injury by targeting WTAP/ATF4 to eliminate ER stress.

Taken together, the present study revealed a new role of WTAP, showing that WTAP promotes myocardial I/R injury through promoting ER stress and cell apoptosis by regulating m^6^A modification of ATF4 mRNA (Figure 6F).

## MATERIALS AND METHODS

### Cell culture and treatment

Human cardiomyocytes (AC16) cells obtained from ATCC (Rockville, MD) were maintained in DMEM with 10% FBS (Thermo Fisher, Bridgewater, NJ). To mimic hypoxia conditions, cells were cultivated in serum-free DMEM and cultured under 5% CO_2_, 1% O_2_, and 94% N_2_ for two hours. Then, cells were cultured under normal condition for 12, 24, and 48 h to mimic reperfusion.

### Transfection

To overexpress WTAP or ATF4, their coding sequences were ligated to pLVX-Puro plasmids (OriGene, Rockville, MD). siRNA (Table 2) specific to WTAP, ATF4, or EIF3A was ligated into linearized pLKO.1 plasmid (OriGene, Rockville, MD). Recombinant plasmids, along with psPAX2 and pMD2G packaging plasmids, were transfected in 293T cells using Lipofectamine 2000 (Invitrogen; catalog 11668019). At 48 h after transfection, viruses were collected for transduction. Cells transduced with scramble siRNA (shNC) or blank vector were considered negative controls.

**Table 2:** Interfering RNA sequences used in this study.

**Gene**	**Sequences (5′-3′)**
Human WTAP shRNA#1	GCAAGTACACAGATCTTAA
Human WTAP shRNA#2	GCGAAGTGTCGAATGCTTA
Human WTAP shRNA#3	GGGCAACACAACCGAAGAT
Human ATF4 shRNA#1	GGAGATCCAGTACCTGAAA
Human ATF4 shRNA#2	GATCCAGTACCTGAAAGAT
Human ATF4 shRNA#3	TGATAGAAGAGGTCCGCAA
Human EIF3A shRNA#1	GCGCCTGTACCATGATATT
Human EIF3A shRNA#2	GCGAGTCACAAAGGTTCTA
Human EIF3A shRNA#3	GCGATCATCCTGGCGTAAT

### Cell apoptosis assay

AC16 cells (50% confluence) were maintained with Annexin V-FITC for twenty minutes in the dark, followed by incubation with PI for twenty minutes. FACScan flow cytometry (Becton Dickinson, Franklin Lakes, NJ) with Cell Quest software (Becton Dickinson) was then performed to examine apoptosis of cells.

### Quantitative real-time PCR (qRT-PCR)

RNAs were extracted with TRIzol (Invitrogen, Waltham, MA). qRT-PCR was performed on an ABI 7000 cycler with SYBR qPCR mix reagent (Bio-Rad, Philadelphia, PA). Primer sequences were shown in Table 3. GAPDH was the internal control. The 2^-ΔΔCT^ formula was used to calculate gene expression.

**Table 3:** Primes sequences used in this study.

**Gene**	**Sequences (5′-3′)**
METTL3-forward	CCTTTGCCAGTTCGTTAGTC
METTL3-reverse	TCCTCCTTGGTTCCATAGTC
METTL14-forward	CTGGGAATGAAGTCAGGATAG
METTL14-reverse	CCAGGGTATGGAACGTAATAG
WTAP-forward	AAAGCAGTGAGTGGGAAAG
WTAP-reverse	AGCGGCAGAAGTATTGAAG
KIAA1429-forward	GCCCTCTTCCACCATTAC
KIAA1429-reverse	ACCACTGCCTCCACTAAC
ATF4-forward	TACAACTGCCCTGTTCCC
ATF4-reverse	GCTGAATGCCGTGAGAAG
EIF3A-forward	GCTCTGGATGTTCTTTATG
EIF3A-reverse	GCTGAGATTCTTCTTTAGC
GAPDH-forward	AATCCCATCACCATCTTC
GAPDH-reverse	AGGCTGTTGTCATACTTC

### Western blot analysis

Total proteins were resolved using 8–10% SDS-PAGE, transferred onto PVDF membranes, and blocked using 3% BSA dissolved in TBST (Tris-buffered saline, 0.1% Tween 20) for 2 h at room temperature. The membranes were incubated with primary antibodies against WTAP (Abcam; catalog ab195380; 1:1000), METTL3 (Abcam; catalog ab195352; 1:1000), METTL14 (Abcam; catalog ab220030; 1:500), KIAA1429 (Proteintech, catalog 25712-1-AP; 1:500), Cleaved PARP (Abcam; catalog ab32064; 1:1000), Cleaved Caspase-3 (Abcam; catalog ab2302; 1:500), p-PERK (Biorbyt; catalog orb191598; 1:1000), PERK (Abcam; catalog ab65142; 1:500), p-eIF2α (Cell Signaling Technology; catalog #9721; 1:1000), eIF2α (Cell Signaling Technology; catalog #5324; 1:1000), ATF4 (Cell Signaling Technology; catalog #11815; 1:1000),CHOP (Cell Signaling Technology; catalog #2895; 1:1000), EIF3A (Abcam; catalog ab128996; 1:1000), and GAPDH (Abcam; catalog ab9485; 1:1000), then incubated with secondary antibodies (Beyotime; catalog A0208, catalog A0216)Protein bands were quantified using Image J.

### m^6^A analysis

m^6^A levels were measured using m^6^A RNA Methylation Assay Kit (Abcam; catalog ab185912) following manufacturer’s protocol.

### RNA immunoprecipitation (RIP) assays

RIP was performed using the Magna RIP RNA-Binding Protein Immunoprecipitation kit (Millipore; catalog 17-700). RNA was isolated, reverse-transcribed, and used for qRT-PCR.

### Protein stability assay

To evaluate protein stability, AC16 cells transduced with EIF3A shRNA vector were treated with 100 μg/ml cycloheximide (CHX, Merck Millipore, Germany; catalog 508739) for different time courses and harvested. Then protein level of ATF4 was determined by western blot analysis.

### Reporter gene assay

ATF4 5′UTR sequence was inserted to pGl3 vector (Promega, Madison, WI). AC16 cells treated with H/R and transduced with WTAP shRNA or overexpression vector were co-transfected with pGl3-ATF4 5′UTR plasmid. AC16 cells treated with H/R and transduced with ATF4 shRNA vector were co-transfected with pGL3-basic plasmid containing the 5′-promoter region of WTAP and the pRL-TK vector.

### Chromatin immunoprecipitation (ChIP) assay

Cells were fixed with 1% formaldehyde, harvested, sonicated, and incubated with anti-ATF4 (Abcam; catalog 184909; 1:50) or control antibody (Proteintech; catalog 30000-0-AP; 1:50) for 12 hours. ATF4 binding was measured using PCR with following primers: 5′-TAAGGAAAGACTACACTAT-3′ and 5′-TGTATTTTTAATAGAGATG-3′.

### Animal experiments

Cardiac I/R was established in 10 week-old male SD rats (Charles River, Wilmington, MA) as described previously [32]. Rats were anesthetized, intubated and mechanically ventilated. Left anterior descending coronary artery (LAD) was ligated for 20 minutes, followed by 48 h reperfusion. Controls underwent same procedures except LAD ligation. WTAP shRNA vector or its negative control (shNC) was injected into the left ventricular anterior wall 24 h before I/R. A pressure volume catheter (Millar Instruments, Houston, TX) was used for cardiac function assay [32]. Echocardiograms were recorded using a Vevo 770 high-resolution ultrasound imaging system (FUJIFILM VisualSonics, Toronto, ON, Canada) equipped with a dedicated micro-visualization scan-head probe (RMV-707B, single element probe) [33]. Rats were then euthanized and myocardium were collected for hematoxylin and eosin (HE) staining or TUNEL assay [34]. Animal Experimentation Ethics Committee of the Seventh People’s Hospital of Shanghai University of Traditional Chinese Medicine approved all experiments performed in this study.

### Data analysis

Data are expressed as the mean ± standard deviation (SD) using GraphPad Prism 8.0.2. Statistical significances were measured using unpaired Student’s *t* tests, one-way, or two-way analysis of variance. *P* values < 0.05 were defined as statistically significant.

## Supplementary Material

Supplementary Figures
