# Supplementary material for: WTAP promotes myocardial ischemia/reperfusion injury by increasing endoplasmic reticulum stress via regulating m6A modification of ATF4 mRNA
Source: Aging (Albany NY). 2021 Mar 26;13(8):11135–49. doi: 10.18632/aging.202770 (PMC8109143; doi:10.18632/aging.202770)
Supplement: Supplementary Figures [file aging-13-202770-s001.pdf]

SUPPLEMENTARY FIGURES

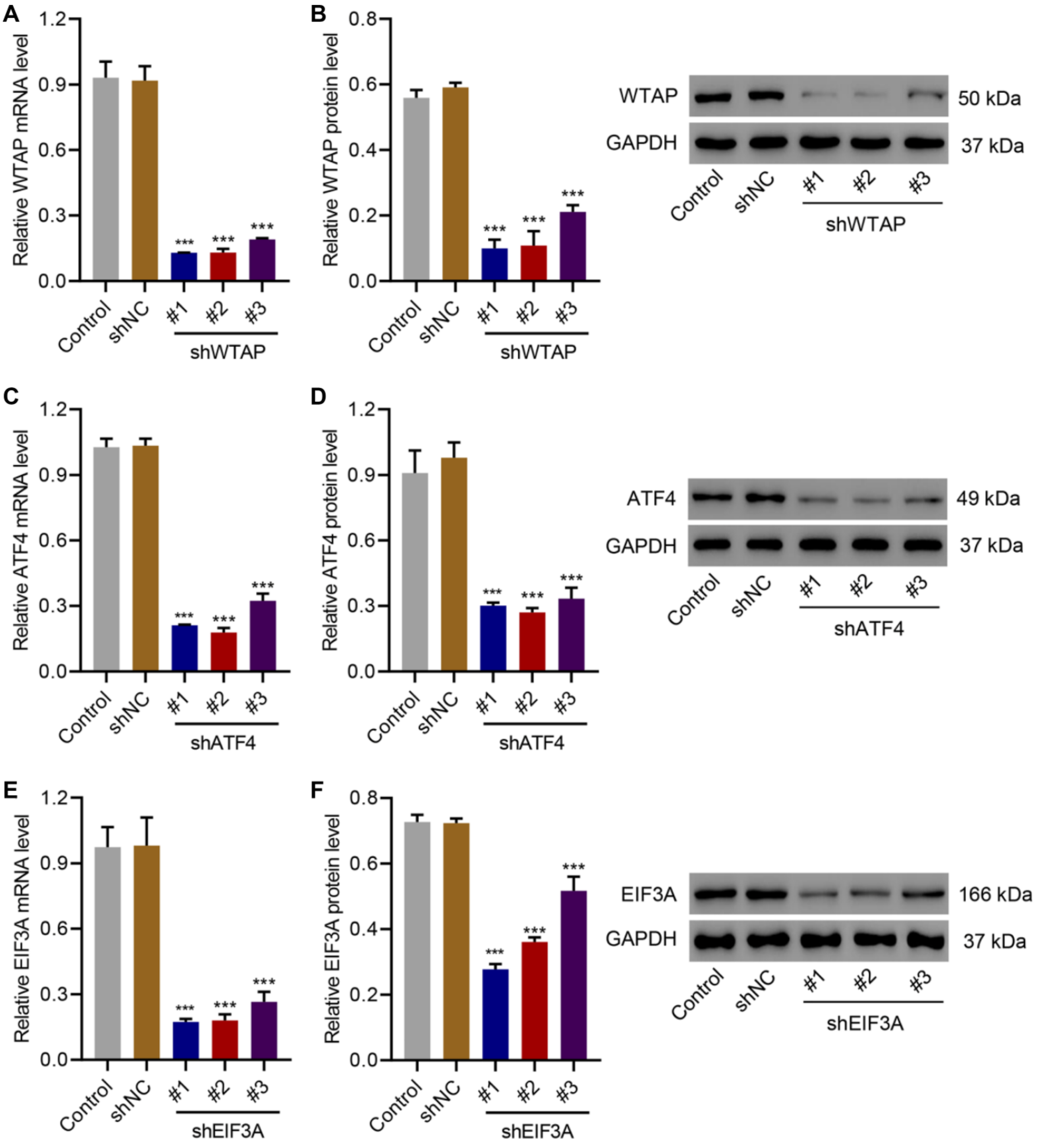

Supplementary Figure 1. Knockdown of WTAP, ATF4, or EIF3A in AC16 cells. Relative mRNA and protein levels of (**A, B**) WTAP, (**C, D**) ATF4, and (**E, F**) EIF3A. All experiments were repeated at least three times, and data are represented as mean ± SD. $^{***}P < 0.001$ compared with shNC.

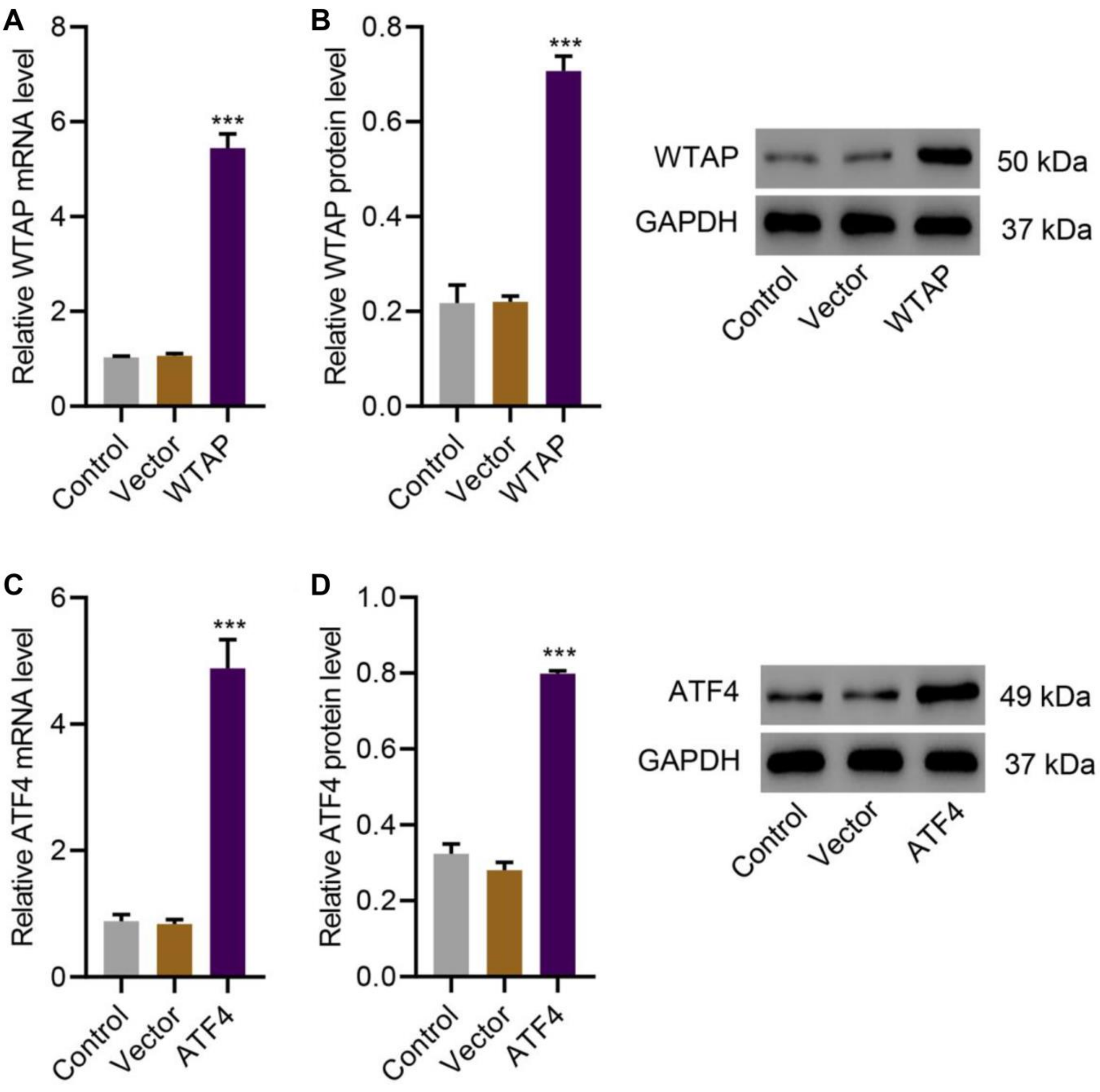

Supplementary Figure 2. WTAP or ATF4 overexpression in AC16 cells. Relative mRNA and protein levels of (**A, B**) WTAP and (**C, D**) ATF4. All experiments were repeated at least three times, and data are represented as mean ± SD. $^{***}P < 0.001$ compared with vector.
